# Targeted temperature management in patients with severe heatstroke: Three case reports and treatment recommendations

**DOI:** 10.1097/MD.0000000000023159

**Published:** 2020-11-06

**Authors:** Yoon Seok Jung, Hyuk-Hoon Kim, Hee Won Yang, Sangchun Choi

**Affiliations:** Department of Emergency Medicine, Ajou University School of Medicine, Suwon, Republic of Korea.

**Keywords:** complications, heat stroke, hypothermia, induced, multiple organ failure

## Abstract

**Rationale:**

Unprecedented heatwaves over the past several years are getting worse with longer duration in the course of global warming. Heatstroke is a medical emergency with multiple organ involvement and life-threatening illness with a high mortality rate of up to 71%. Uncontrolled damage to the central nervous system can result in severe cerebral edema, permanent neurological sequelae, and death. However, regarding the therapeutic aspects of heat stroke, there was no therapeutic strategy after the rapid cooling of the core body temperature to <39°C to prevent further injury.

**Patient concerns:**

Each of 3 patients developed a change of mental statuses after the exposure to summer heatwaves or relatively high environmental temperatures with high humidity in the sauna.

**Diagnoses:**

The patients were diagnosed with severe heatstroke since they showed cerebral edema and multiple organ dysfunction based on the results from laboratory tests and the findings in brain computed tomography scan.

**Interventions:**

The patients underwent induced therapeutic hypothermia (<36°C) between 24 and 36 hours in the management of severe heatstroke.

**Outcomes:**

The patients survived from cerebral edema and multiple organ dysfunction.

**Lessons:**

We believe that targeted temperature management (<36°C) will help treat severe heatstroke. Thus it should be considered for reducing the chance of development of complications in multiple organs, especially in the central nervous system, when managing patients with severe heatstroke.

## 1 Introduction

As the intensity and duration of an unprecedented heatwave over the past several years are rising in the course of global warming, populations exposed to the risk of heat-related illnesses are increasing.^[1–4]^ Heat-related illnesses can be categorized based on the severity of symptoms. Heatstroke, characterized by a core body temperature that rapidly rises above 40°C and is associated with hot, dry skin and central nervous system (CNS) dysfunction, causes multiple-organ severe complications, including permanent neurological sequelae.^[5]^ Although the mechanism of heatstroke is not fully understood, several theoretical and experimental models have described how systemic complications and neurological sequelae—especially cerebellar dysfunctions that are common in neurological sequelae—are caused by direct thermal injury and sepsis-like responses in heatstroke. Severe heatstroke, which involves exposure to higher temperatures for more extended periods, may progress to multiple organ failure and death. In such cases, severe cerebral edema can result in permanent neurological sequelae or death.^[6]^ Moreover, according to Hifumi et al,^[6]^ the mortality rate is up to 71%. However, regarding the therapeutic aspects of heat stroke, there was no therapeutic strategy after the rapid cooling of the core body temperature to <39°C to prevent further injury. Therapeutic hypothermia improved survival and reduced the rate of disability in patients with cardiac arrest.^[7]^ We propose a novel method of inducing therapeutic hypothermia (<36°C) between 24 and 36 hours in the management of heatstroke patients to prevent neurological sequelae caused by heatstroke. Based on the consideration of physiological changes in hypothermia and the pathophysiology of heatstroke, a more aggressive body temperature control than the previous cooling method of just falling below 39°C should be instituted in treating heatstroke, which is similar to therapeutic hypothermia (<36°C) for 24 hours that is applied routinely in post-cardiac arrest patients. The implementation of such guidelines could result in favorable prognoses in patients with heatstroke.

## 2 Cases

There were three patients of heatstroke managed with induced hypothermia in this paper. Ajou institutional review board approved a waiver of informed consent based on its official rule of a waiver of informed consent for a case report of <5 patients. All patients provided written consent for publication.

### 2.1 Methodology of targeted temperature management

Our targeted temperature management (TTM) protocol was: achieving the target temperature between 32°C and 36°C and then maintaining for >24 hours unless the patient is hemodynamically unstable, has a bleeding tendency, or has an ongoing severe infection. TTM was induced using ice packs, intravenous cold saline, and cooling devices that include a feedback-controlled surface cooling device system (Blanketrol II, Cincinnati Subzero Products, Cincinnati, OH; Artic Sun Energy Transfer Pads, Medivance Corp., Louisville, KY). The core temperature of a patient was monitored using an esophageal or foley catheter temperature probe. Remifentanil, dexmedetomidine, and propofol were used for sedation and analgesia. During the maintenance phase, the constant targeted temperature was maintained. Upon the completion of the maintenance phase, patients were rewarmed with the target rate of 0.25°C/h. Advanced critical care, such as oxygenation, ventilation, glucose control, and hemodynamic optimization, was provided according to guidelines. All patients had sufficient sedation and analgesia as needed for shivering control and seizure prevention.

### 2.2 Case A

**Figure 1:**
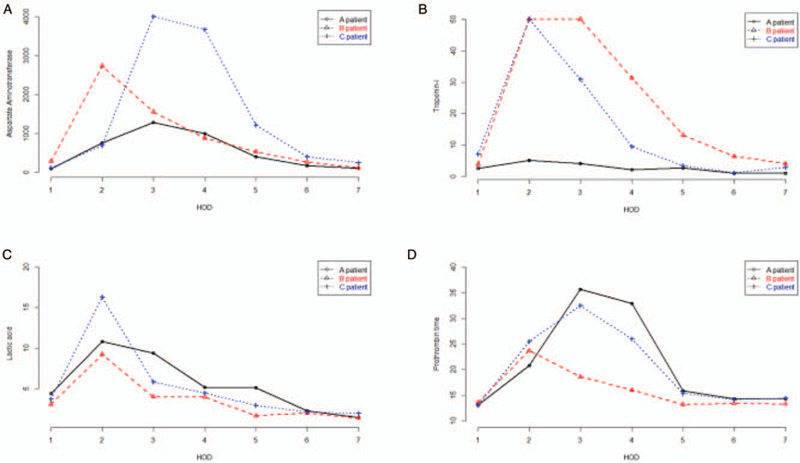
Changes in laboratory results after hospital admission. (A) Aspartate aminotransferase, (B) Troponin-I, (C) Lactic acid, (D) Prothrombin time. Laboratory test results, representative of systemic organs, increased rapidly out of normal range after admission to the ICU, even though the initial heat insult was removed from patients. Then, all values of laboratory results rose to the highest between hospital days 2 and 4. Targeted temperature management might weaken further injuries subject to initial heat insult in multiple organs.

A 73-year-old female was admitted to the emergency department (ED) complaining of mental change. It was a hot summer day with an atmospheric temperature of about 34°C, but there was no air conditioning system in the patient's house except for an electric fan. The vital signs were: blood pressure 106/51 mmHg, pulse rate 160 bpm, respiratory rate 41 cpm, body temperature 40.1°C, and pulse oxygen saturation 92%. Endotracheal intubation was performed at the time of the ED arrival. Simultaneously, ice-pack application, evaporative cooling of water spray and fanning over the skin, and cold fluid administration were conducted. In the history of the patient, she had hypertension, diabetes mellitus, and asthma. She was alert until 10 pm on the previous day. She was found to have a fever, rapid respiration, sweating, and decreased mentality in the morning. On physical examination, there were large amounts of redness and small bullae on the dependent positions of her back, extremities, buttocks. A brain computed tomography (CT) scan was performed, but nothing specific to her conditions was found. There was no definite focus for high body temperature except heat-related illness. Although her body core temperature decreased to 38°C, she did not regain her consciousness. Induced hypothermia of 34 °C was performed for the next 24 hours. She regained consciousness after the TTM was conducted, and she was discharged on hospital day 73 with some disability of movement. Changes in laboratory results were shown in Figure 1.

### 2.3 Case B

A 61-year-old male was admitted to the ED because he was found unconscious inside the sauna in a public bathhouse. No one knew when he entered the sauna. The vital signs were: blood pressure 40/30 mmHg, pulse rate 150 bpm, respiratory rate 30 cpm, and body temperature 40.1°C. Both of his pupils were pinpoint size. On physical examination, his mentality was semicomatose. Crackles were heard in both lung fields, and there were skin abrasions secondary to contact burns in the dependent portions of both elbows. Rapid sequence intubation was performed. Simultaneously, ice-pack application, evaporative cooling of water spray and fanning over the skin, and cold fluid administration were conducted. Although 2 L of crystalloid fluid was administered, systolic blood pressure remained around 70 mmHg; therefore, norepinephrine was administered at a rate of 2.5 μg/min/kg. His body temperature fell to around 38°C after 50 minutes had passed since his admission to the ED. He was not responsive to painful stimuli 2 hours after his body temperature had dropped to <38°C. An emergency brain CT scan did not show any hemorrhage or low HU lesions, except cerebral swelling. Therefore, we conducted TTM (32°C –34°C) for 36 hours, and he was admitted to the ICU. After termination of TTM, he showed a tiny response to external stimuli; however, sudden cardiac arrest occurred. Cardiopulmonary resuscitation was performed for 4 minutes on hospital day of 8. On hospital day of 11, he opened the eyes spontaneously. On hospital day of 46, he was discharged, although his extremities had disabilities (cerebral performance categories scale [CPC] 3). Changes in laboratory results were shown in Figure 1.

### 2.4 Case C

A 21-year-old male was transferred to the ED because he had severe heatstroke during a troop march in hot weather. In the presenting illness, he became unconscious while marching with a backpack weight of 50 pounds. He was admitted to the military hospital immediately, where his vital signs were blood pressure 70/40 mmHg, pulse rate 200 rpm, respiratory rate 46 rpm, and body temperature 42.2°C. There was no specific finding in the brain and cervical spine computed tomography (CT) scan. Laboratory test values were within normal except for CPK, which was elevated to 2000 IU/L. Based on his laboratory tests, there was no evidence of infectious disease. He was transferred to our ED because he showed unconsciousness 4 hours later. His mentality varied from deep stupor to semicomatose. His vital signs were: BP 146/91 mmHg, PR 140 rpm, RR 50 rpm, and BT 38.4°C upon his admission to the ED. Simultaneously, ice-pack application, evaporative cooling of water spray and fanning over the skin, and cold fluid administration were conducted for lowering body temperature. His body temperature fell to <38°C after 20 minutes had passed since his admission to the ED. He was still unresponsive to painful stimuli 2 hours after his body temperature had fallen <38°C.

The emergency brain CT scan did not show any hemorrhage or low HU lesions, except moderate cerebral swelling. Therefore, we conducted TTM (<36°C) for 36 hours. He was admitted to the ICU, where he underwent continuous renal replacement therapy because he showed acute kidney injury—hypercreatinekinasemia. At the time of TTM termination, he showed good responses to verbal stimuli. At hospital day of 3, he was extubated, although there was a small pneumonic consolidation in the right middle lobe. There was no deficit regarding his neurological system function. He was transferred once again to the military hospital on hospital day 5 and was discharged with no neurological deficit. Changes in laboratory results were shown in Figure 1.

## 3 Discussion

The diagnosis of heatstroke is made by ruling out other diagnoses to identify the one that explains the patient's conditions the best. In our study, patients were diagnosed with heatstroke if they had been exposed to a hot environment and suffered from a change of mental statuses relating to increased body temperature with no definitive evidence for the existence of different disease entities. Previous studies have examined the possible role of active and passive cooling to treat heatstroke. Although rapid cooling of the core body temperature is the essential therapeutic aspect when treating heatstroke, there was no therapeutic strategy to implement after rapid cooling and falling of the core body temperature. Untreated or uncontrolled systemic injury, including CNS damage secondary to the heatstroke, resulted in severe CNS injuries, multiple organ failure, and death. As showed in Figure 1, laboratory results, representative of systemic organs, increased rapidly out of normal range, even though the initial heat insult was removed from the patients.

TTM, previously known as therapeutic hypothermia or induced hypothermia, is an active treatment for achieving and maintaining specific body temperatures of a patient for a set time duration of time.^[7,8]^ The goal of TTM is to improve neurologic outcomes during the recovery phase after the ischemic/anoxic period.^[7,8]^ TTM may reduce the risk of ischemia-reperfusion injury, and it is the first safe and effective therapy for ischemia-reperfusion injuries in cardiac arrest and stroke.^[7,8]^ Previous literature reported similar neurologic outcomes for the target temperature of 33°C (91°F) to 36°C (97°F).^[7]^ Complications, including infection and bleeding, may occur in TTM, which is thought to play a role in the prevention of brain injury by reducing the brain's oxygen demands, the production of neurotransmitters (eg, glutamate), and free radicals that can damage the brain.

**Figure 2:**
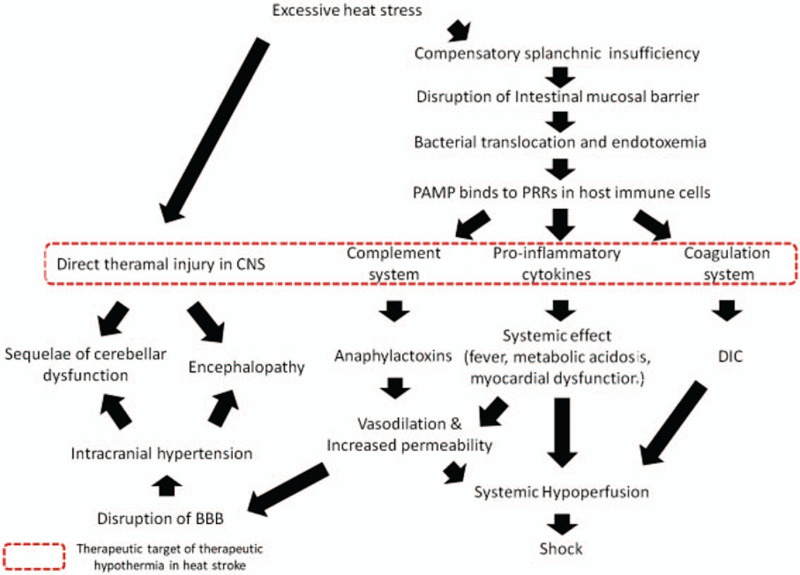
Pathophysiology of heatstroke and target of therapeutic-induced hypothermia. Targeted temperature management could prevent multiple-organ failure, including CNS injury, from aggravating in patients with heatstroke because it could reduce the overwhelming immunologic responses and direct thermal injury to the CNS in the dotted box.

We expected that TTM would assist in the treatment of patients with heatstroke by alleviating the assumed mechanisms of heatstroke: thermal-direct injury, sepsis-like responses, diminishing neuronal injury, and the mitigation of inflammatory responses and immune responses (Fig. 2).

### 3.1 Thermal-direct injury

Thermal-direct injury to cells and tissues starts at temperatures >41.7°C in mammals.^[9]^ In extreme temperatures above the threshold of the accommodation response, cellular membranes liquefy, enzymes denature or modify and lose their functions, and mitochondrial oxidative phosphorylation is altered and disrupted by uncoupling reactions.^[5,6]^ Furthermore, the generation of reactive oxygen species (ROS)—a critical mediator of heat stress-induced apoptosis—is provoked. Additionally, the ROS-dependent mitochondrial signaling pathway is associated with activated apoptosis in hyperthermia.^[10]^ Heat adversely affects almost all organ systems, with the CNS being particularly vulnerable. In the CNS, the cerebellum is the most susceptible to this thermal-direct injury, followed by the cerebral cortex, brainstem, and spinal cord.^[11]^ Several cases and postmortem studies have reported selective vulnerability of the cerebellum, showing localized swelling of Purkinje cells and cell death, with the duration of hyperthermia correlating with the extent of cell death.^[12]^ Furthermore, systemic sepsis-like reactions in heatstroke induce additional injuries to the CNS by disrupting the blood-brain barrier, leading to an accumulation of inflammatory cells in the entire CNS, including thermal-direct injured tissues.^[13]^ Cytotoxic edema after thermal-direct neuronal injury and vasogenic edema followed by the disruption of the blood-brain barrier induce intracranial hypertension, which may exacerbate the neurological injuries.^[14]^ As a result, encephalopathy and cerebellar syndromes, such as truncal ataxia, severe dysarthria, dysmetria, and dysdiadochokinesia, are frequently prominent in patients with heatstroke even after normalization of the body temperature and recovery from the acute phase of heatstroke. Therefore, neuroprotective strategies should be implemented to reduce the occurrences of neurological sequelae in managing heatstroke.

### 3.2 Sepsis-like responses

During exposure to heat, which may be from high environmental temperature (classic or non-exertional) or strenuous exercise (exertional), the thermoregulatory center in the hypothalamus strictly maintains the core temperature at approximately 37°C by dissipating the heat load through cutaneous vasodilation and increasing blood flow in the skin in humans. Despite this adaptation process, thermoregulation failure combined with an exaggerated acute inflammatory response results in the progression of heat stress to heatstroke.^[15]^ During vigorous efforts to shift the heated blood from core circulation to peripheral circulation, inevitable shunting effects lead to circulatory insufficiency in the gastrointestinal (GI) system.^[16]^ Reduction of splanchnic blood flow incurs hypoxic/ischemic injuries of the hepatocellular and GI system, which consequently induces cell membrane damage and dissociation of a cell-to-cell junction in the GI mucosa, thus adversely affecting cell viability and cell permeability.^[16]^ Alteration of immunologic and barrier functions of the intestines allows leakage of endotoxins into the systemic circulation.^[15]^ In a persistent hyperthermic status, which cannot be compensated properly, endotoxin spillover overwhelms the hepatic capacity of detoxification and may trigger the initial inflammatory reaction in heatstroke.^[15,17]^

The immune response to endotoxemia resulting from the collapse of the intestinal mucosal barrier activates the signaling cascade originating from pattern recognition receptors such as toll-like receptors in host immune cells in heatstroke.^[18]^ Interleukin-1 and tumor necrosis factor-α, the potent inflammatory mediators, play an essential role in the initial immune response. Both cytokines can cause fever, hypotension, leukocytosis, induction of other proinflammatory cytokines, and simultaneous activation of coagulation and fibrinolysis.^[19]^ As tissue factors—one of the critical mediators of disseminated intravascular coagulation—are released in response to exposure to cytokines and endotoxin, the dysregulation of processes participating in coagulation and fibrinolysis is also provoked in heatstroke.^[20]^ The activation of coagulation coincides with the onset of heatstroke, as assessed by the appearance of thrombin–antithrombin III complexes, soluble fibrin monomers, and low levels of protein C, protein S, and antithrombin III. Fibrinolysis is also activated, as shown by increased levels of plasmin and d-dimers and decreased levels of plasminogen.^[5]^ The complement system, which helps in the clearance of pathogens from organisms and plays a significant role in immune responses, is also activated in heat stroke, leading to the release of peptides, which are potent mediators of inflammatory and immune responses known as anaphylatoxins. These peptides bind to their respective receptors on immune cells to initiate inflammation and vasodilation, which activates them.^[5,15]^

Heatstroke is also defined as a type of hyperthermia associated with a systemic inflammatory response. The molecular pathways and clinical features of aggressive inflammatory reactions in heatstroke correspond with the processes in sepsis (Fig. 1).^[5]^ These sepsis-like responses in heatstroke induce additional injuries to the CNS by disrupting the blood-brain barrier, leading to an accumulation of inflammatory cells in the entire CNS, including thermal-direct injured tissues. The causes of death in heatstroke closely resemble the end-stage clinical features of sepsis, which are respiratory failure, acute respiratory distress syndrome, nosocomial pneumonia, renal failure, GI bleeding, stress gastritis, anemia, deep venous thrombosis, electrolyte abnormalities, hyperglycemia, hepatic dysfunction, and disseminated intravascular coagulation.^[21]^ Consequently, bacterial translocation and endotoxemia from the GI system caused by systemic insults associated with heat stress lead to a systemic inflammatory response that mimics sepsis, and that can deteriorate rapidly to multiple organ failure and death.^[15]^

### 3.3 Diminishing neuronal injury subject to heatstroke

Therapeutic hypothermia has protective effects against thermal-direct neuronal injury. The severity and extent of thermal-direct injury depend on the critical thermal maximum, a term that attempts to qualify the level and duration that will initiate tissue injury.^[22]^ Since the extent of tissue damage is determined by the degree of heat, and the duration of exposure, lower duration of exposure to hyperthermia through rapid induction of hypothermia results in less neuronal tissue damage.

Following a period of thermal-direct injury, cells and tissues of the penumbra around the tissue going through irreversible necrotic changes after primary damage may be fully or partially recovered, but may also enter the path leading to apoptosis.^[10,14]^ Whether apoptosis will develop is determined by cellular processes such as mitochondrial dysfunction, which blocks cellular energy metabolism; release of caspase enzymes, which plays an essential role in the process; and disequilibrium of intracellular ion (especially Ca^2+^) homeostasis, which induces prolonged opening of the mitochondrial permeability transition pore and is related to cellular survival in heatstroke.^[10]^ Numerous studies have shown that hypothermia mainly affects these critical factors by preventing mitochondrial dysfunction, inhibition of caspase activation, and modification of intracellular Ca^2+^ ion concentration.^[23,24]^ These cytoprotective effects of hypothermia may prevent the surviving neuronal tissues (after initial thermal-direct injury) from undergoing apoptosis and help reduce neurological sequelae.

In addition, therapeutic hypothermia reduces the disruption of the blood-brain barrier and reduces vascular permeability, further reducing cerebral edema formation.^[25]^ These effects of hypothermia may help alleviate the exacerbation of intracerebral hypertension after thermal-direct injury and lead to a favorable neurological prognosis in heatstroke.

### 3.4 Mitigation of inflammatory and immune response subject to heatstroke

Therapeutic hypothermia, which targets lower-than-normal body temperature, may lead to beneficial effects through the inhibition and mitigation of the complex immune responses in heatstroke. Numerous animal experiments and some clinical studies have shown that hypothermia reduces the release of proinflammatory cytokines and coincidently stimulates the secretion of anti-inflammatory cytokines such as interleukin-10.^[26,27]^ In addition, hypothermia can impair neutrophil and macrophage function and reduce a leukocytosis reaction.^[28]^ Therapeutic hypothermia could also modulate the complement system by controlling the expression of complement fragments, especially C3a and C5a.^[29]^ Furthermore, mild hypothermia exerts an anticoagulant effect, which may have inhibitory effects on the microthrombus formation and inhibit fibrinolysis and platelet activation during cooling.^[30]^

Given the likelihood that many processes are activated simultaneously with the complex pathogenesis of heat stroke, the combined inhibition of the many aspects of the inflammatory process would provide the breakthrough therapy anticipated. Currently, there is no other treatment of heatstroke but to lower the initial temperature. However, complicated mechanisms are involved in heatstroke, which necessitates additional treatments for improved prognosis. Here, TTM may play a crucial role in elucidating complicated mechanisms involved in heatstroke.

## 4 Conclusions

Most patients recover well after a period of hyperthermia, but patients exposed to higher temperatures for more extended periods are more at risk of complications, which in extreme cases may progress to multiple organ failure, especially the CNS, and death. As aforementioned, the reported mortality is as high as 71%. Current treatments for heatstroke do not adequately control the underlying mechanisms of injuries; thus, the effectiveness of these treatments remains unsatisfactory and questionable. Therefore, we think that the application of TTM of <36°C for 24 to 36 hours, which can control multifactorial pathophysiological processes, helps treat patients with severe heatstroke as a fundamental therapeutic modality.

## Author contributions

**Conceptualization:** Sangchun Choi.

**Data curation:** Hyuk-Hoon Kim.

**Formal analysis:** Hee Won Yang.

**Investigation:** Yoon Seok Jung, Hee Won Yang.

**Supervision:** Sangchun Choi.

**Writing – original draft:** Hyuk-Hoon Kim.

**Writing – review & editing:** Yoon Seok Jung, Sangchun Choi.

## References

[1] Torjesen I Heat related deaths could rise from 2000 to 12000 a year by the 2080 s, health agency says. BMJ 2012;345:e6138. 22968862 10.1136/bmj.e6138

[2] Saleem SG Ansari T Ali AS Risk factors for heat related deaths during the June 2015 heat wave in karachi, Pakistan. J Ayub Med Coll Abbottabad 2017;29:320–4. 28718257

[3] Qiao Z Guo Y Yu W Assessment of short- and long-term mortality displacement in heat-related deaths in Brisbane, Australia, 1996-2004. Environ Health Perspect 2015;123:766–72. 25794410 10.1289/ehp.1307606 PMC4529002

[4] Centers for Disease Control and Prevention. Heat-related deaths after an extreme heat event--four states, 2012, and United States, 1999-2009. MMWR Morb Mortal Wkly Rep 2013;62:433–6. 23739336 PMC4604981

[5] Bouchama A Knochel JP Heat stroke. N Engl J Med 2002;346:1978–88. 12075060 10.1056/NEJMra011089

[6] Hifumi T Kondo Y Shimizu K Heat stroke. J Intensive Care 2018;6:30. 29850022 10.1186/s40560-018-0298-4 PMC5964884

[7] Nielsen N Wetterslev J Cronberg T Targeted temperature management at 33°C versus 36°C after cardiac arrest. N Engl J Med 2013;369:2197–206. 24237006 10.1056/NEJMoa1310519

[8] Kim YM Park KN Choi SP Part 4. Post-cardiac arrest care: 2015 Korean Guidelines for Cardiopulmonary Resuscitation. Clin Exp Emerg Med 2016;3: suppl: S27–38. 27752644 10.15441/ceem.16.130 PMC5052921

[9] Shibolet S Lancaster MC Danon Y Heat stroke: a review. Aviat Space Environ Med 1976;47:280–301. 769777

[10] Gu ZT Li L Wu F Heat stress induced apoptosis is triggered by transcription-independent p53, Ca(2+) dyshomeostasis and the subsequent Bax mitochondrial translocation. Sci Rep 2015;5:11497. 26105784 10.1038/srep11497 PMC4478470

[11] Mahajan S Schucany WG Symmetric bilateral caudate, hippocampal, cerebellar, and subcortical white matter MRI abnormalities in an adult patient with heat stroke. Proc (Bayl Univ Med Cent) 2008;21:433–6. 18982090 10.1080/08998280.2008.11928446 PMC2566920

[12] Ookura R Shiro Y Takai T Diffusion-weighted magnetic resonance imaging of a severe heat stroke patient complicated with severe cerebellar ataxia. Intern Med 2009;48:1105–8. 19525609 10.2169/internalmedicine.48.2030

[13] Kosgallana AD Mallik S Patel V Heat stroke induced cerebellar dysfunction: a “forgotten syndrome”. World J Clin Cases 2013;1:260–1. 24340279 10.12998/wjcc.v1.i8.260 PMC3856304

[14] Polderman KH Mechanisms of action, physiological effects, and complications of hypothermia. Crit Care Med 2009;37: 7 suppl: S186–202. 19535947 10.1097/CCM.0b013e3181aa5241

[15] Epstein Y Roberts WO Golan R Sepsis, septic shock, and fatal exertional heat stroke. Curr Sports Med Rep 2015;14:64–9. 25574888 10.1249/JSR.0000000000000112

[16] Lambert GP Role of gastrointestinal permeability in exertional heatstroke. Exerc Sport Sci Rev 2004;32:185–90. 15604939 10.1097/00003677-200410000-00011

[17] Sakurada S Hales JR A role for gastrointestinal endotoxins in enhancement of heat tolerance by physical fitness. J Appl Physiol (1985) 1998;84:207–14. 9451637 10.1152/jappl.1998.84.1.207

[18] Cinel I Dellinger RP Advances in pathogenesis and management of sepsis. Curr Opin Infect Dis 2007;20:345–52. 17609592 10.1097/QCO.0b013e32818be70a

[19] Pruitt JH Copeland EM 3rd Moldawer LL Interleukin-1 and interleukin-1 antagonism in sepsis, systemic inflammatory response syndrome, and septic shock. Shock 1995;3:235–51. 7600191 10.1097/00024382-199504000-00001

[20] van der Poll T Tissue factor as an initiator of coagulation and inflammation in the lung. Crit Care 2008;12: suppl 6: S3. 10.1186/cc7026 PMC2607113 19105796

[21] Chun JK Choi S Kim HH Predictors of poor prognosis in patients with heat stroke. Clin Exp Emerg Med 2019;6:275–81. 10.15441/ceem.18.081 PMC6952628 31910506

[22] Hutchison VH The concept of critical thermal maximum. Am J Physiol 1979;237:R367–368. 495788 10.1152/ajpregu.1979.237.5.R367

[23] Xu L Yenari MA Steinberg GK Mild hypothermia reduces apoptosis of mouse neurons in vitro early in the cascade. J Cereb Blood Flow Metab 2002;22:21–8. 11807390 10.1097/00004647-200201000-00003

[24] Liou AK Clark RS Henshall DC To die or not to die for neurons in ischemia, traumatic brain injury and epilepsy: a review on the stress-activated signaling pathways and apoptotic pathways. Prog Neurobiol 2003;69:103–42. 12684068 10.1016/s0301-0082(03)00005-4

[25] Chi OZ Liu X Weiss HR Effects of mild hypothermia on blood-brain barrier disruption during isoflurane or pentobarbital anesthesia. Anesthesiology 2001;95:933–8. 11605935 10.1097/00000542-200110000-00023

[26] Kimura A Sakurada S Ohkuni H Moderate hypothermia delays proinflammatory cytokine production of human peripheral blood mononuclear cells. Crit Care Med 2002;30:1499–502. 12130969 10.1097/00003246-200207000-00017

[27] Sipos W Duvigneau C Sterz F Changes in interleukin-10 mRNA expression are predictive for 9-day survival of pigs in an emergency preservation and resuscitation model. Resuscitation 2010;81:603–8. 20163907 10.1016/j.resuscitation.2010.01.014

[28] Dufner MC Andre F Stiepak J Therapeutic hypothermia impacts leukocyte kinetics after cardiac arrest. Cardiovasc Diagn Ther 2016;6:199–207. 27280083 10.21037/cdt.2016.02.06 PMC4880752

[29] Gong P Zhao H Hua R Mild hypothermia inhibits systemic and cerebral complement activation in a swine model of cardiac arrest. J Cereb Blood Flow Metab 2015;35:1289–95. 25757755 10.1038/jcbfm.2015.41 PMC4528002

[30] Gong P Zhang MY Zhao H Effect of mild hypothermia on the coagulation-fibrinolysis system and physiological anticoagulants after cardiopulmonary resuscitation in a porcine model. PLoS One 2013;8:e67476. 23818980 10.1371/journal.pone.0067476 PMC3688589

